# Synthesis and Dual Histamine H_1_ and H_2_ Receptor Antagonist Activity of Cyanoguanidine Derivatives

**DOI:** 10.3390/molecules181114186

**Published:** 2013-11-15

**Authors:** Bassem Sadek, Rudi Alisch, Armin Buschauer, Sigurd Elz

**Affiliations:** 1 Department of Pharmacology and Therapeutics, College of Medicine and Health sciences, UAE University, Al-Ain, P.O. Box 17666, UAE; 2 Institute of Pharmacy, Free University of Berlin, Königin-Luise-Str. 2-4, Berlin D-14195, Germany; 3 Institute of Pharmacy, University of Regensburg, University Str. 31, Regensburg D-93053, Germany

**Keywords:** dual H_1_/H_2_ receptor antagonists, mepyramine, roxatidine, tiotidine, ranitidine

## Abstract

Premedication with a combination of histamine H_1_ receptor (H_1_R) and H_2_ receptor (H_2_R) antagonists has been suggested as a prophylactic principle, for instance, in anaesthesia and surgery. Aiming at pharmacological hybrids combining H_1_R and H_2_R antagonistic activity, a series of cyanoguanidines **14**–**35** was synthesized by linking mepyramine-type H_1_R antagonist substructures with roxatidine-, tiotidine-, or ranitidine-type H_2_R antagonist moieties. *N*-desmethylmepyramine was connected via a poly-methylene spacer to a cyanoguanidine group as the “urea equivalent” of the H_2_R antagonist moiety. The title compounds were screened for histamine antagonistic activity at the isolated ileum (H_1_R) and the isolated spontaneously beating right atrium (H_2_R) of the guinea pig. The results indicate that, depending on the nature of the H_2_R antagonist partial structure, the highest H_1_R antagonist potency resided in roxatidine-type compounds with spacers of six methylene groups in length (compound **21**), and tiotidine-type compounds irrespective of the alkyl chain length (compounds **28**, **32**, **33**), *N*-cyano-*N'*-[2-[[(2-guanidino-4-thiazolyl)methyl]thio]ethyl]-*N″*-[2-[*N*-[2-[*N*-(4-methoxybenzyl)-*N*-(pyridyl)-amino] ethyl]-*N*-methylamino]ethyl] guanidine (**25**, p*K*_B_ values: 8.05 (H_1_R, ileum) and 7.73 (H_2_R, atrium) and the homologue with the mepyramine moiety connected by a six-membered chain to the tiotidine-like partial structure (compound **32**, p*K*_B_ values: 8.61 (H_1_R) and 6.61 (H_2_R) were among the most potent hybrid compounds. With respect to the development of a potential pharmacotherapeutic agent, structural optimization seems possible through selection of other H_1_R and H_2_R pharmacophoric moieties with mutually affinity-enhancing properties.

## 1. Introduction

The biogenic amine histamine mediates its effects via four histamine receptor subtypes, termed H_1_, H_2_, H_3_, and H_4_ receptors (H_x_R) [1,2,3]. The histamine receptors belong to class A of the superfamily of G-protein-coupled receptors (GPCRs). As an autacoid and neurotransmitter, histamine is involved in numerous physiological and pathophysiological processes. Antagonists of the H_3_R [4] and the most recently discovered H_4_R [5] are still under investigation as potential drugs, e.g., for the treatment of CNS disorders and inflammatory diseases, respectively [6]. By contrast, H_1_R and H_2_R antagonists are well established therapeutic agents for decades. Numerous pathophysiological responses to histamine released from mast cells and basophils through immunological or non-immunological mechanisms are mediated by the H_1_R, for instance, vasodilatation via nitric oxide release, increase in capillary permeability, contraction of smooth muscles, e.g., in gut and bronchi. The first H_1_R blockers [7] have been described as “antihistamines” more than 70 years ago, and especially the newer non-sedating H_1_R antagonists are widely used in the treatment of allergic conditions [7]. Stimulation of gastric acid secretion is the most prominent physiological effect of H_2_R stimulation. In the 1970s the development of the H_2_R antagonists revolutionized the treatment of gastric and duodenal ulcers [8]. Histamine can also induce cardiovascular effects via H_2_Rs, e.g., increase in heart rate and cardiac contractility as well as vasodilatation [9].

The combined administration of H_1_R and H_2_R antagonists has been suggested, for example, as a prophylactic principle in anaesthesia, for the prevention of hypersensitivity reactions to drugs, e.g., in cancer chemotherapy, or for the treatment of skin diseases [10,11,12,13,14,15]. Histamine release during anaesthesia and surgery is still a widely known problem in clinical practice [10]. The ratio of anaphylactic/anaphylactoid reaction incidents is about 20%–30%, and life threatening reactions are observed in 0.1%–0.5% of all cases [16]. Cardiac arrhythmias induced by activation of H_1_ and H_2_ receptors are among the most serious consequences of mast cell degranulation. Several studies have shown the effectiveness of a premedication with a combination of H_1_R and H_2_R antagonists, for example dimetindene plus cimetidine [10,17,18,19,20].

**Figure 1:**
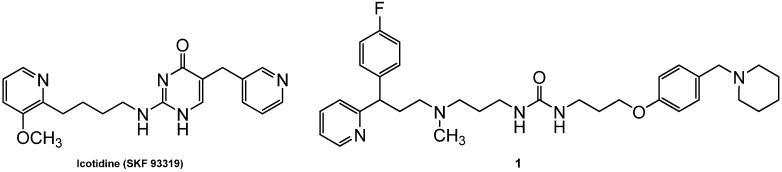
Icotidine (SK&F 93319) and lead compound **1**.

The commercially available H_1_ and H_2_ antihistamines differ considerably concerning physicochemical and pharmacokinetic properties. A combination of both qualities of action in one hybrid drug could be of therapeutic advantage, for instance, in terms of pharmacokinetics. Such hybrid antihistamines could also be of potential interest in other indications, e.g., in the therapeutic management of atopic dermatitis. Icotidine (SK&F 93319), derived from the isocytosine series of H_2_R antagonists, was the first substance reported to possess nearly equipotent antagonist activity at both H_1_R and H_2_R [21] (Figure 1).

Previously, urea-type H_1_/H_2_ receptor antagonists such as compound **1** were synthesized and pharmacologically investigated [22,23,24]. Lessons learned, in particular from the H_4_R and H_3_R, suggest that differences between H_x_R species orthologues should to be taken into account [2,3]. Nevertheless, isolated guinea-pig organs have been used as standard pharmacological models to characterize H_1_R and H_2_R antagonists for many decades, and the data gained from these investigations provided a reliable basis for numerous successfully marketed histamine receptor antagonists. Therefore, with respect to potential studies in translational animal models, the synthesized compounds were investigated on the guinea pig isolated ileum and the spontaneously beating right atrium. The urea derivative **1**, containing a three-membered carbon chain as a spacer and fluoropheniramine substructure, had a p*K*_B_ value of 8.21 at the isolated guinea pig ileum and proved to be more active than pheniramine at the H_1_R. However, H_2_R antagonist activity (guinea pig atrium: p*K*_B_ 6.68) was found to be moderate, presumably due to introduction of a basic centre close to the polar group [23] (Figure 1).

**Figure 2:**
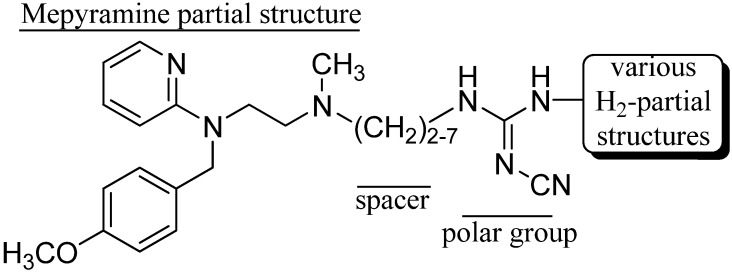
General structure of spacer-linked combined H_1_/H_2_ receptor antagonists.

As mepyramine (pA_2_ 9.07 ± 0.03) [25] is more potent than pheniramine-like H_1_R antagonists, in the present study, the mepyramine partial structure was used as a building block for the synthesis of hybrid compounds. The H_1_R antagonist pharmacophoric moiety was connected via a carbon chain (spacer) of variable length and a cyanoguanidine group with the H_2_R antagonist partial structure of roxatidine, tiotidine, and ranitidine (Figure 2). Thereby, the cyanoguanidine moiety, a characteristic “urea equivalent” [26,27] of H_2_R antagonists such as cimetidine, was kept unchanged as a bioisosteric replacement of the polar moieties present in roxatidine, tiotidine and ranitidine, respectively.

## 2. Results and Discussion

### 2.1. Chemistry

**Scheme 1:**
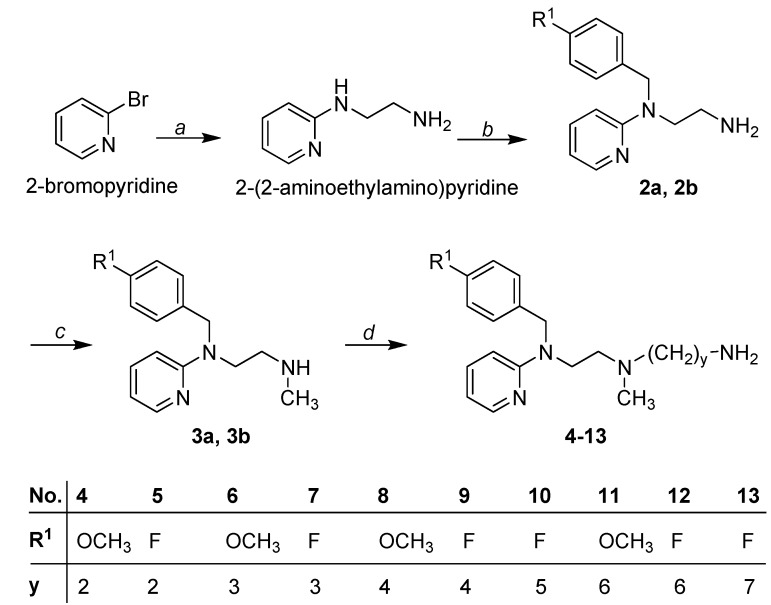
Synthetic pathway for intermediates **4**–**13**.

The cyanoguanidines **14**–**35** were synthesized starting from triamines **4**–**13** which can be easily prepared from the secondary amines **3a** and **3b** through alkylation with acrylonitrile or *ω*-haloalkanenitriles of different chain lengths followed by reduction with LiAlH_4_ in diethyl ether (Scheme 1). Secondary amines **3a** and **3b** were obtained by acylation of primary amines **2a** and **2b** with ethyl chloroformate and subsequent reduction with LiAlH_4_ in tetrahydrofuran [23,24].

*Reagents and Conditions*: ^*a*^ Ethylenediamine, pyridine, reflux, 3 h; ^*b*^ NaH, 4-methoxybenzylchloride (for **2a**), 4-fluorobenzylchloride (for **2b**), DMSO, room temperature, 12 h; ^*c*^ NaOH, ethyl chloroformate, THF, room temperature; ^*d*^ *(i)* Acrylonitrile or corresponding ω-chloroalkylnitrile, KI, excess Na_2_CO_3_, 60 °C, 2 h; *(ii)* Lithium aluminum hydride (50% excess), diethyl ether, room temperature, 2 h.

**Scheme 2:**
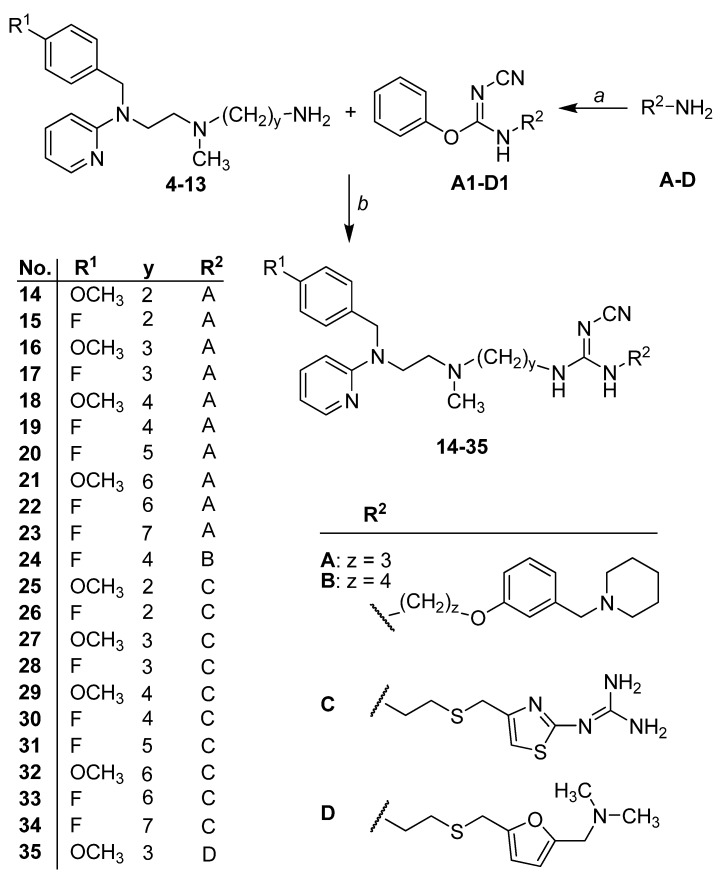
Synthetic pathway of cyanoguanidines **14**–**35**.

The triamines **4**–**13** were allowed to react with the isourea intermediates **A1**–**D1** (Scheme 2) to yield the cyanoguanidines **14**–**35**[22,23,24,28,29,30,24,28]. Addition of the roxatidine-like primary amines **A** and **B** resulted in **14**–**23** and **24**, respectively, while addition of **C** and **D** afforded the compounds **25**–**34** and **35**, respectively [24].

### 2.2. Pharmacology

The synthesized hybrid compounds **14**–**35** were investigated for histamine H_1_R and H_2_R antagonism at the isolated guinea pig ileum (H_1_R) and the isolated spontaneously beating guinea pig right atrium (H_2_R). The results are summarized in Table 1.

*Reagents and Conditions*: *^a^* Diphenyl *N*-cyanocarbonimidate, room temperature, 2 h; *^b^* Respective diamine, **A1**-**D1**, acetonitrile, reflux, 16 h.

Compounds **14**–**35** showed histamine H_1_R antagonistic activities on guinea pig ileum with p*K*_B_ values in the range from 6.8 (compound **14**) to 8.6 (compounds **28**, **32**). Although the activity increased with the length of the polymethylene spacer in case of the methoxy-substituted derivatives bearing a roxatidine-like H_2_-antagonist moiety (compound **14*** vs.* **16**, **18** and **21**), a clear general tendency was not obvious, neither for the corresponding fluorinated analogues nor for the guanidinothiazoles **25**–**34**. Highest H_1_R antagonistic activity resided in the latter group of compounds. Though an affinity-increasing effect was not evident, it should be noted that the interactions with the H_1_R were not dramatically affected in an adverse manner by the H_2_R antagonist moiety. The most potent H_1_R antagonists achieved *K*_B_ values in the one-digit nanomolar range corresponding to about 30% of the activity of mepyramine at the guinea-pig ileum.

**Table 1:** Histamine H_1_ and H_2 _receptor antagonism of cyanoguanidines **14**–**35**. 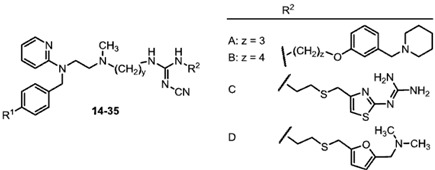

				H_1_ Receptor Antagonism			H_2_ Receptor Antagonism		
Compound	R^1^	y	R^2^	p *K*_B_^a^	rel. activity	conc. [M]	p *K*_B_^a^	rel. activity	conc. [M]
Mepyramine	-	-	-	9.07 ^b^	100		-		
Cimetidine	-	-	-	-			6.40	100	
Icotidine ^c^	-	-	-	7.77	5.0		7.49	1230	
**1** ^d^				8.21	14	0.3 × 10^−6^	6.68	190	10^−6^–0.3 × 10^−5^
**14**	OCH_3_	2	A	6.88	1	0.3 × 10^−6^	6.63	170	10^−7^–0.3 × 10^−6^
**15**	F	2	A	7.83	6	10^−7^	5.97 ^e^	37	0.3 × 10^−5^
**16**	OCH_3_	3	A	7.81	6	10^−7^–0.3 × 10^−6^	6.37	93	0.3 × 10^−6^
**17**	F	3	A	7.77 ^e^	5	10^−7^–0.3 × 10^−6^	6.0	40	0.3 × 10^−6^–10^−6^
**18**	OCH_3_	4	A	7.76	5	10^−8^–10^−7^	6.38	96	0.3 × 10^−6^
**19**	F	4	A	7.75	5	10^−7^–0.3 × 10^−7^	5.76 ^e^	23	10^−5^–0.3 × 10^−5^
**20**	F	5	A	7.95	7	10^−7^	5.67	19	0.3 × 10^−5^
**21**	OCH_3_	6	A	8.42	22	10^−7^–0.3 × 10^−7^	6.43	107	0.3 × 10^−6^
**22**	F	6	A	8.06	10	10^−7^–0.3 × 10^−7^	6.41	102	10^−6^
**23**	F	7	A	7.93	7	10^−7^–0.3 × 10^−7^	6.17	59	10^−6^–0.3 × 10^−5^
**24**	F	4	B	7.65	4	10^−7^–0.3 × 10^−6^	6.07	47	10^−6^
**25**	OCH_3_	2	C	8.05	10	10^−7^–0.3 × 10^−7^	7.37	933	10^−7^
**26**	F	2	C	7.94	7	10^−8^–0.3 × 10^−7^	6.62	166	10^−6^
**27**	OCH_3_	3	C	8.21	14	10^−8^–0.3 × 10^−7^	6.52	132	10^−6^
**28**	F	3	C	8.62	36	10^−8^–0.3 × 10^−8^	6.47	118	0.3 × 10^−6^–0.3 × 10^−5^
**29**	OCH_3_	4	C	7.91	7	0.3 × 10^−7^	7.00	398	10^−7^–0.3 × 10^−6^
**30**	F	4	C	8.32	18	10^−8^–0.3 × 10^−8^	6.28	76	10^−6^
**31**	F	5	C	8.36	20	10^−8^–0.3 × 10^−7^	6.51	129	10^−6^–10^−5^
**32**	OCH_3_	6	C	8.61	35	10^−7^–0.3 × 10^−7^	6.61	162	10^−7^–0.3 × 10^−6^
**33**	F	6	C	8.44	23	0.3 × 10^−7^	6.52	132	10^−6^
**34**	F	7	C	8.15	12	10^−8^	7.02	417	10^−6^–0.3 × 10^−6^
**35**	OCH_3_	3	D	7.95	7	10^−8^–10^−7^	5.22 ^e^	7	10^−5^

^a^ Determined at the isolated ileum (H_1_) and right atrium (H_2_) of the guinea pig; p*K*_B_ mean values, S.E.M. for ileum within ±0.2 by **16**, **17**, **18**, and **19**; ±0.1 by all other compounds; p*K*_B_ mean values, S.E.M. for atrium within ±0.2 by **14**, and **17**; ±0.1 by all other compounds; rel. activity, % activity relative to mepyramine, icotidine or cimetidine, respectively; antag. conc., antagonist concentrations used; ^b^ [25]; ^c^ [21]; ^d^ [23]; ^e^ At 0.3 × 10^−6^ M, depression of the concentration response curve by 15%, 30%, 15%, and 20% for **15**, **17**, and **19**, respectively.

It is well known from numerous investigational and commercially available H_2_R antagonists (e.g., cimetidine, ranitidine or tiotidine) that, comparing the numeric values, the highest antagonistic activities achievable at the guinea pig right atrium (Table 1) are usually by one to two orders of magnitude lower than at the guinea pig ileum. The same holds for the investigated hybrid compounds. Highest H_2_R antagonistic activity resided in guanidinothiazole **25** with a p*K*_B_ value of 7.37, corresponding to nine-fold higher activity than cimetidine or three-fold lower activity than tiotidine, respectively. Among the cyanoguanidines substituted with roxatidine-like partial structures (compounds **14**–**23**), lengthening of the spacer from ethylene to pentamethylene decreased both antagonist activities on H_1_ and H_2_ receptors. However, cyanoguanidine derivative **21** (p*K*_B_ ileum, 8.42, p*K*_B_ atrium, 6.43) with a hexamethylene spacer separating the basic center from the polar cyanoguanidine moiety was approximately two times more active on H_1 _receptors, and the H_2_ antagonistic activity was in the same range as that of reference compounds **1** or cimetidine. Moreover, the presence of a methoxy group at *p*-position of the mepyramine partial structure seemed to be of advantage over fluorine substitution. Compared to **22**, the H_1_R antagonist activity of **21** was approximately twofold higher whereas the H_2_R antagonist remained unchanged. Further extension of the spacer up to seven methylene groups in the H_1_R antagonist partial structure or up to four methylene groups in the H_2_R antagonist moiety decreased both H_1_R and H_2_R antagonistic activities as demonstrated by cyanoguanidine derivatives **23** (p*K*_B_ ileum, 7.93, p*K*_B_ atrium, 6.17) and **24** (p*K*_B_ ileum, 7.65, p*K*_B_ atrium, 6.07), respectively. Thus, compound **21** represents the optimum in terms of combined H_1_R/H_2_R antagonistic activity among the piperidinomethylphenoxyalkylcyanoguanidines.

A further increase in potency was achieved by replacement of the roxatidine-like moiety with a guanidinothiazole (compounds **25**–**34**) as in tiotidine, resulting in the highest histamine H_1_R and H_2_R antagonistic activities among this series of pharmacological hybrids. Compound **32** with a hexamethylene spacer was found to be up to two times more potent at both H_1_R and H_2_R compared to the roxatidine-like analog **21**. In addition, the results revealed that a *p*-methoxy moiety (compound **32**), unlike *p*-fluoro substituent (compound **33**), potentiates the antagonistic activities at both H_1_ and H_2_ receptors. Remarkably, further extension of the spacer up to 7 methylene groups resulted in cyanoguanidine derivative **34**, the most potent H_2_R antagonist amid the title compounds. However, H_1_R antagonistic decreased up to 5-fold compared to compounds **32** and **33**. The cyanoguanidine derivative **35**, bearing a ranitidine-like moiety, did not show an activity-enhancing effect compared to the corresponding parent compounds. Compound **25** with an ethylene spacer and tiotidine partial structure revealed the highest H_2_R antagonistic activity among the presented series of compounds.

In summary, the combination of the mepyramine substructure with a tiotidine-like guanidinothiazole turned out to be most suitable to obtain pharmacological hybrid with activities comparable to those of commercially available selective H_1_R and H_2_R antagonists.

## 3. Experimental

### 3.1. Chemistry

#### 3.1.1. General Conditions

Melting points are uncorrected and determined in open capillaries in a Buechi 512 Dr. Tottoli apparatus. ^1^H-NMR spectra were recorded on a Bruker WC 300 spectrometer with tetramethylsilane (TMS) as internal standard. Chemical shifts are reported in ppm downfield from internal tetramethylsilane as reference. ^1^H-NMR signals are reported in order: multiplicity (s, single; d, doublet; t, triplet; q, quintet; m, multiplet; br, broad; *, exchangeable by D_2_O), number of protons, and approximate coupling constants in Hertz. Elemental analyses were performed on Perkin-Elmer 240B and 240C instruments. Analyses (C, H, N) indicated by the symbols of elements were within ±0.4% of the theoretical values. Chromatographic separations were done by rotation planar chromatography (centrifugal layer chromatography) using a Chromatotron Model 7924 (Harrison Research, Muttenz, Switzerland) with 4-mm layers of silica gel 60 PF_254_ containing gypsum (Merck). To avoid tailing, a saturated ammonia atmosphere was produced by passing through a stream of anhydrous gaseous ammonia via the “inert gas inlet” of the Chromatotron. EI-mass spectra were recorded using Finnigan MAT CH7A (70 eV), Finnigan MAT 711 (80 eV), or Kratos MS 25 RF (70 eV). ^+^FAB-MS spectra were recorded on Finnigan MAT CH5DF instrument (xenon, DMSO)/glycerol). The following abbreviations are used: DMF, *N*,*N*-dimethylformamide,; EtOH, ethanol; Et_2_O, diethyl ether; MeOH, methanol; Me_2_SO, dimethyl sulfoxide; Ph, phenyl; * exchangeable with D_2_O; C_2_H_2_O_4_, oxalic acid; Py, pyridyl; Pip, piperidyl; TA, C_4_H_6_O_6_, tartaric acid; C_20_H_18_O_8_, (−)-di-p-tolyltartaric acid; decomp, decomposition.

#### 3.1.2. Synthesis of Primary Amines **2a** and **2b**

2-Bromopyridine (109.30 mmol) and ethylenediamine (546 mmol) were refluxed in pyridine for 3 h. Excess of ethylenediamine was evaporated under reduced pressure, water was added, the pH value was adjusted to >11 using NaOH (1N), and the 2-(2-aminoethylamino)-pyridine was extracted with chloroform [29]. After evaporation of chloroform, the product was purified by means of a Chromatotron (chloroform/methanol, 97/3, V/V, ammonia atmosphere), and used in the following reaction without further purifications. The primary amines **2a** and **2b** were synthesized by suspending 2-(2-aminoethylamino)pyridine (73.40 mmol) with sodium hydride (60% oil suspension, 64.50 mmol) in DMSO under nitrogen atmosphere. The reaction temperature was increased gradually and kept at 85 °C until hydrogen evolution ceased. For synthesis of **2a** and **2b**, 4-methoxybenzylchloride and 4-fluorobenzylchloride, respectively, were added, and the mixture was stirred for 12 h at room temperature. Subsequently, water (500 mL) was added, **2a** and **2b** were extracted with diethyl ether at pH > 11, combined extracts were dried over anhydrous potassium carbonate, and the solvent was evaporated under reduced pressure yielding the oily products **2a** and **2b**, which were used in the following reaction to prepare **3a** and **3b **without further purification.

#### 3.1.3. Synthesis of Secondary Amines **3a** and **3b**

For synthesis of **3a** and **3b**, 43.64 mmol of the corresponding primary amine **2a** and **2b** were dissolved in diethyl ether, NaOH (10%) was added and the solution was kept over ice-bath. Ethyl chloroformate was added gradually to the two-phase system. After complete reaction, the diethyl ether layer was separated, dried over sodium sulfate, and evaporated under reduced pressure to yield the oily products **3a** and **3b**, respectively [22,23,24,28].

#### 3.1.4. General Procedure for the Synthesis of the Diamines **4**–**13**

As previously described, a mixture of the corresponding secondary amine **3a** or **3b** (10–30 mmol), an equimolar amount of the pertinent *ω*-halonitrile, a catalytic amount of potassium iodide and a 100% excess of sodium carbonate was stirred for 2 h at 60 °C in 20 mL of a mixture of acetonitrile and dimethylformamide (1:1 (V/V) in case of chloroacetonitrile, 9:1 (V/V) in case of homologous halonitriles) [22,23,24,28]. Subsequently, water was added, the mixture was extracted with toluene, the combined extracts were dried over sodium sulfate and evaporated under reduced pressure yielding the corresponding oily aminonitriles as intermediates, which were dissolved in 20 mL of anhydrous diethyl ether and dropped to an ice-cold suspension of lithium aluminium hydride (50% excess). After 2 h stirring at room temperature the reaction mixture was hydrolyzed by addition of water followed by 3 mL of a 10% aqueous sodium hydroxide solution. The resulting diamines **4**–**13** were purified chromatographically (Chromatotron, eluent: chloroform/methanol, gradient from 99:1 to 90:10 (V/V), ammonia atmosphere), and were used in the following reactions for synthesis of cyanoguanidines **14**–**35**[22,23,24,28].

#### 3.1.5. General Procedure for the Synthesis of Cyanoguanidines **14**–**35**

The pertinent *N*-cyano *O*-phenyl isoureas (2–4 mmol) **A1**–**D1**, which were prepared by stirring of equivalent amounts of **A**–**D** with diphenyl *N*-cyanocarbonimidate for 2 h, and equimolar amounts of the respective diamines **4**–**13** in 30 mL of anhydrous acetonitrile, were heated under reflux for 16 h. The mixture was evaporated to dryness and the cyanoguanidines **14**–**35** were isolated chromatographically (Chromatotron, chloroform: methanol, gradient from 99:1 to 90:10, ammonia atmosphere) [22,23,24,28].

*N-Cyano-N'-[2-[N-[2-[N-(4-methoxybenzyl)-N-(2-pyridyl)amino]ethyl]-N-methylamino]ethyl]-N″-[3-[3-(piperidinomethyl)phenoxy]propyl]guanidine* (**14**). Yield: 65%, mp: 88–89 °C; ^1^H-NMR (CDCl_3_):δ ppm = 1.41–1.42 (m, 2H, PipH); 1.53–1.57 (m, 4H, PipH), 1.98 (m, 2H, CH_2_); 2.25 (s, 3H, N-CH_3_); 2.34 (br, 4H, PipH); 2.57–2.62 (m, 4H, 2(CH_2_-NCH_3_)); 3.20–3.21 (m, 2H, CH_2_-NH); 3.30–3.32 (m, 2H, CH_2_-NH); 3.40 (s, 2H, Ph-CH_2_- Pip); 3.66 (t, *J* = 7, 2H, CH_2_-N); 3.76 (s, 3H, Ph-OCH_3_); 3.97 (t, *J* = 6, 2H, Ph-O-CH_2_); 4.60 (s, 2H, Ph-CH_2_-N); 5.85 (br, 1H*, NH); 6.40-6.43 (d, *J* = 9, 1H aromatic, Py5-H); 6.51–6.55 (m, 1H aromatic, Py3-H); 6.73–6.75 (d, *J* = 8.5, 1H aromatic, 4-H); 6.81–6.84 (d, *J* = 8, 2H aromatic, 2-H); 6.87-6.90 (d, *J* = 8, 2H aromatic, 2-H); 7.10–7.13 (m, 2H aromatic, 3-H); 7.10–7.13 (br, 1H*, NH); 7.17 (t, *J* = 8, 1H aromatic, 3-H); 7.36 (m, 1H aromatic,Py4-H); 8.12–8.13 (d, *J* = 3, 1H aromatic, Py6-H); MS: *m/z* (%) 612 (M^+^, 1), 121 (100); Anal. Calcd. for C_35_H_48_N_8_O_2_・H_2_O: C, 66.60; H, 7.99; N, 17.80. Found: C, 66.70; H, 7.82; N, 17.45.

*N-Cyano-N'-[2-[N-[2-[N-(4-fluorobenzyl)-N-(2-pyridyl)amino]ethyl]-N-methylamino]ethyl]-N″-[3-[3-(piperidinomethyl)phenoxy]propyl]guanidine* (**15**). Yield: 33%, mp: 106–108 °C; ^1^H-NMR (CDCl_3_): δ ppm = 1.44 (m, 2H, PipH); 1.61 (m, 4H, PipH), 1.90 (m, 2H, CH_2_); 2.31 (s, 3H, N-CH_3_); 2.59–2.64 (br, 4H, PipH); 2.71 (m, 4H, 2(CH_2_-NCH_3_)); 3.24 (m, 2H, CH_2_-NH); 3.37–3.47 (m, 2H, CH_2_-NH); 3.63 (m, 2H, CH_2_-N); 3.83 (s, 2H, Ph-CH_2_-Pip); 3.96 (t, 2H, Ph-O-CH_2_); 4.18 (s, 6H, TA); 4.72 (s, 2H, Ph-CH_2_-N); 5.20–5.60 (br, 6H*, TA-OH); 6.54 (m, 2H aromatic, Py5-H, Py3-H); 6.86–6.88 (m, 3H aromatic, 4-H, 2-H); 6.86–6.88 (br, 3H*, 3(H-(N^+^)); 6.92 (m, 2H aromatic, 2-H); 6.92 (br, 1H*, NH); 7.11 (m, 2H aromatic, 3-H); 7.25 (m, 2H aromatic, 3-H); 7.25 (br, 1H*, NH); 7.45 (m, 1H aromatic, Py4-H); 8.06 (d, 1H aromatic, Py6-H); 10.50 (br, 3H*, TA-COOH); MS: *m/z* (%) 601 (M^+^, 7), 76 (100); Anal. Calcd. for C_34_H_45_FN_8_O・3C_4_H_6_O_6_: C, 52.60; H, 6.04; N, 10.70. Found: C, 52.50; H, 6.27; N, 10.60.

*N-Cyano-N'-[3-[N-[2-[N-(4-methoxybenzyl)-N-(2-pyridyl)amino]ethyl]-N-methylamino]propyl]-N″-[3[3-piperidinomethyl)phenoxy]propyl]guanidine* (**16**). Yield: 51%, mp: 62–65 °C; ^1^H-NMR (CDCl_3_): δ ppm = 1.41–1.42 (m, 2H, PipH); 1.53–1.57 (m, 4H, PipH), 1.66 (m, 2H, CH_2_); 2.25 (s, 3H, N-CH_3_); 2.34 (br, 4H, PipH); 2.57–2.62 (m, 4H, 2(CH_2_-NCH_3_)); 3.20–3.21 (m, 2H, CH_2_-NH); 3.30–3.32 (m, 2H, CH_2_-NH); 3.40 (s, 2H, Ph-CH_2_- Pip); 3.66 (t, *J* = 7, 2H, CH_2_-N); 3.76 (s, 3H, Ph-OCH_3_); 3.97 (t, *J* = 6, 2H, Ph-O-CH_2_); 4.60 (s, 2H, Ph-CH_2_-N); 5.85 (br, 1H*, NH); 6.40–6.43 (d, *J* = 9, 1H aromatic, Py5-H); 6.51–6.55 (m, 1H aromatic, Py3-H); 6.73–6.75 (d, *J*=8.5, 1H aromatic, 4-H); 6.81–6.84 (d, *J* = 8, 2H aromatic, 2-H); 6.87–6.90 (d, *J* = 8, 2H aromatic, 2-H); 7.10–7.13 (m, 2H aromatic, 3-H); 7.10–7.13 (br, 1H*, NH); 7.17 (t, *J* = 8, 1H aromatic, 3-H); 7.36 (m, 1H aromatic, Py4-H); 8.12–8.13 (d, *J* = 3, 1H aromatic, Py6-H); MS: *m/z* (%) 627 (M^+^, 6), 241 (11), 121 (100); Anal. Calcd. for C_36_H_50_N_8_O_2_: C, 69.0; H, 8.04; N, 17.90. Found: C, 68.80; H, 8.27; N, 17.70.

*N-Cyano-N'-[3-[N-[2-[N-(4-fluorobenzyl)-N-(2-pyridyl)amino]ethyl]-N-methylamino]propyl]-N″-[3-[3-(piperidinomethyl)phenoxy]propyl]guanidine* (**17**). Yield: 57%, mp: 65–68 °C; ^1^H-NMR (CDCl_3_): δ ppm = 1.43 (m, 2H, PipH); 1.57–1.61 (m, 4H, PipH); 1.68 (m, 2H, CH_2_); 1.99–2.05 (m, 2H, CH_2_); 2.28 (s, 3H, N-CH_3_); 2.40 (br, 4H, PipH); 2.51 (t, *J* = 7, 2H, CH_2_-NCH_3_); 2.58 (t, *J* = 7, 2H, CH_2_-N-CH_3_); 3.25–3.26 (m, 2H, CH_2_-NH); 3.39 (m, 2H, CH_2_-NH); 3.45 (s, 2H, Ph-CH_2_-Pip); 3.70 (t, *J* = 7.5, 2H, CH_2_-N); 4.03 (t, *J* = 6, 2H, Ph-O-CH_2_); 4.63 (s, 2H, Ph-CH_2_-N); 6.41 (d, *J* = 9, 1H aromatic, Py5-H); 6.55 (t, *J* = 6, 1H aromatic, Py3-H); 6.75–6.78 (d, *J* = 8, 1H aromatic, 4-H); 6.89-6.92 (d, *J* = 6, 2H aromatic, 2-H); 6.89–6.92 (br, 1H*, NH); 6.97 (t, *J* = 8, 2H aromatic, 2-H), 7.13–7.21 (m, 2H aromatic, 3-H, 3-H); 7.13–7.21 (br, 1H*, NH); 7.38 (t, *J* = 7, 1H aromatic, Py4-H); 8.12–8.13 (d, *J* = 5, 1H aromatic, Py6-H); MS: *m/z* (%) 615 (M^+^, 14), 229 (100), 109 (96); Anal. Calcd. for C_35_H_47_FN_8_O・3C_4_H_6_O_6_: C, 68.80; H, 8.27; N, 17.70. Found: C, 68.53; H, 8.55; N, 17.21.

*N-Cyano-N'-[4-[N-[2-[N-(4-methoxybenzyl)-N-(2-pyridyl)amino]ethyl]-N-methylamino]butyl]-N″-[3-[3-(piperidinomethyl)phenoxy]propyl]guanidine* (**18**). Yield: 47%, mp: 106–108 °C; ^1^H-NMR (CDCl_3_): δ ppm = 1.44 (m, 4H, PipH); 1.59 (m, 6H, PipH); 1.91 (m, 2H, CH_2_); 2.50 (s, 3H, N-CH_3_); 2.64 (m, 4H, PipH); 2.86 (m, 2H, CH_2_-N-CH_3_); 3.11 (m, 2H, CH_2_-NCH_3_); 3.28 (m, 4H, CH_2_-NH, Ph-CH_2_-Pip); 3.44 (m, 2H, CH_2_-NH); 3.71 (s, 3H, Ph-OCH_3_); 3.75 (m, 2H, CH_2_-N), 3.97 (m, 2H, Ph-O-CH_2_); 4.12 (s, 6H, TA); 4.65 (s, 2H, Ph-CH_2_-N); 6.00 (br, 6H*), TA-OH); 6.55–6.58 (m, 2H aromatic, Py5-H, Py3-H); 6.86–6.88 (m, 3H aromatic, 4-H, 2-H); 6.86–6.88 (br, 4H*), 3 (H-(N^+^)), NH); 6.92–7.00 (m, 2H aromatic, 2-H); 7.06 (br, 1H*, NH); 7.13 (m, 2H aromatic, 3-H); 7.26 (m, 1H aromatic, 3-H); 7.43–7.46 (d, *J* = 7, 1H aromatic, Py4-H); 8.08 (m, 1H aromatic, Py6-H); 10.50 (br, 3H*), TA-COOH); MS: *m/z* (%) 641 (M^+^, 1), 241 (7), 121 (100); Anal. Calcd. for C_37_H_52_N_8_O_2_・3C_4_H_6_O_6_: C, 51.60; H, 6.29; N, 10.20. Found: C, 52.01; H, 6.47; N, 10.30.

*N-Cyano-N'-[4-[N-[2-[N-(4-fluorobenzyl]-N-(2-pyridyl)amino]ethyl]-N-methylamino]butyl]-N″-[3-[3-(piperidinomethyl)phenoxy]propyl]guanidine* (**19**). Yield: 48%, mp: 84–86 °C; ^1^H-NMR (CDCl_3_): δ ppm = 1.37 (m, 2H, CH_2_); 1.46 (m, 2H, CH_2_); (see **15**); MS: *m/z* (%) 629 (M^+^, 5), 2229 (84), 109 (100); Anal. Calcd. for C_36_H_49_FN_8_O・3C_4_H_6_O_6_: C, 53.90; H, 6.73; N, 10.40. Found: C, 51.30; H, 6.46; N, 9.97.

*N-Cyano-N'-[4-[N-[2-[N-(4-methoxybenzyl)-N-(2-pyridyl)amino]ethyl]-N-methylamino]pentyl]-N″-[3-[3-(piperidinomethyl)phenoxy]propyl]guanidine* (**20**). Yield: 17%, mp: 86–88 °C; ^1^H-NMR (CDCl_3_): δ ppm = 1.22 (m, 4H, 2(CH_2_)); (see **17**); MS: *m/z* (%) 643 (M^+^, 7), 229 (54), 154 (100); Anal. Calcd. for C_37_H_51_FN_8_O・3C_4_H_6_O_6_: C, 53.80; H, 6.36; N, 10.30. Found: C, 54.30; H, 6.83; N, 10.30.

*N-Cyano-N'-[6-[N-[2-[N-(4-methoxybenzyl)-N-(2-pyridyl)amino]ethyl]-N-methylamino]hexyl]-N″-[3-[3-(piperidinomethyl)phenoxy]propyl]guanidine* (**21**) Yield: 66%, mp: 79–81 °C; ^1^H-NMR (CDCl_3_): δ ppm = 1.23–1.25 (m, 4H, 2(CH_2_)); 1.42–1.43 (m, 4H, 2(CH_2_)); (see **14**); MS: *m/z* (%) 669 (M^+^, 5), 121 (100); Anal. Calcd. for C_39_H_56_N_8_O_2_·3C_4_H_6_O_6_: C, 54.70; H, 6.66; N, 10.10. Found: C, 54.60; H, 7.01; N, 9.79.

*N-Cyano-N'-[6-[N-[2-[N-(4-fluorobenzyl)-N-(2-pyridyl)amino]ethyl]-N-methylamino]hexyl]-N″-[3-[3-(piperidinomethyl)phenoxy]propyl]guanidine* (**22**). Yield: 35%, mp: >58 °C decomposed; ^1^H-NMR (CDCl_3_): δ ppm = 1.25 (m, 4H, 2(CH_2_)); 1.42 (m, 2H, CH_2_); (see **25**); MS: *m/z* (%) 657 (M^+^, 11), 229 (100); Anal. Calcd. for C_38_H_53_FN_8_O・3C_4_H_6_O_6_: C, 52.50; H, 6.61; N, 9.80. Found: C, 52.70; H, 6.80; N, 9.80.

*N-Cyano-N'-[7-[N-[2-[N-(4-fluorobenzyl)-N-(2-pyridyl)amino]ethyl]-N-methylamino]heptyl]-N″-[3-[3-(4-(piperidin-1-ylmethyl)phenoxy]propyl]guanidine* (**23**). Yield: 41%, mp: 96–98 °C; ^1^H-NMR (CDCl_3_): δ ppm = 1.21-1.25 (m, 6H, 3(CH_2_)); 1.42 (m, 2H, CH_2_); (see **17**); MS: *m/z* (%) 671 (M^+^, 3), 229 (100); Anal. Calcd. for C_39_H_55_FN_8_O・1.5C_20_H_18_O_8_: C, 65.30; H, 6.62; N, 8.83. Found: C, 65.24; H, 6.90; N, 8.65.

*N-Cyano-N'-[4-[N-[2-(4-fluorobenzyl)-N-(2-pyridyl)amino]ethyl]-N-methylamino]butyl)-N″-[3-[4-(4-(piperidin-1-ylmethyl)phenoxy]butyl]guanidine* (**24**). Yield: 37%, mp: 102–104 °C; ^1^H-NMR (CDCl_3_): δ ppm = 1.68-1.75(m, 2H, CH_2_); (see **17**); MS: *m/z* (%) 629 (M^+^, 18), 229 (98), 109 (100); Anal. Calcd. for C_36_H_49_FN_8_O・1.5C_20_H_18_O_8_: C, 64.60; H, 6.36; N, 9.13. Found: C, 64.40; H, 6.51; N, 8.77.

*N-Cyano-N'*-*[2-[[(2-guanidino-4-thiazolyl)methyl]thio]ethyl]-N″-[2-[N-[2-[N-(4-methoxybenzyl)-N-(2-pyridyl)amino]ethyl]-N-methylamino]ethyl]guanidine* (**25**). Yield: 58%, mp: 56–58 °C; ^1^H-NMR (CDCl_3_): δ ppm = 2.23 (s, 3H, N-CH_3_); 2.50 (m, 4H, CH_2_-S, CH_2_-NCH_3_); 2.56 (m, 2H, CH_2_-NCH_3_); 3.16–3.17 (m, 2H, CH_2_-NH); 3.26–3.28 (m, 2H, CH_2_-NH); 3.37 (s, 2H, Thi-CH_2_-S); 3.58 (m, 2H, CH_2_-N); 3.70 (s, 3H, Ph-OCH_3_); 4.66 (s, 2H, Ph-CH_2_-N); 6.45 (s, 1H aromatic., Thi5-H); 6.50–6.54 (m, 2H aromatic, Py5-H, Py3-H);, 6.84–6.87 (m, 2H aromatic, 2-H); 6.84–6.87 (br, 2H*, 2(NH)); 6.94 (br, 4H*, NC(NH_2_)_2_); 7.14 (d, *J* = 8, 2H aromatic, 3-H); 7.41 (m, 1H aromatic, Py4-H); 8.05–8.07 (d, *J* = 4, 1H aromatic, Py6-H); MS: *m/z* (%) 596 (M^+^, 2), 121 (100); Anal. Calcd. for C_27_H_37_N_11_OS_2_・1.5C_2_H_2_O_4_: C, 54.30; H, 6.75; N, 24.0. Found: C, 54.21; H, 6.63; N, 24.11.

*N-Cyano-N'-[2-[[(2-guanidino-4-thiazolyl)methyl]thio]ethyl]-N″-[2-[N-[2-[N-(4-fluoro-benzyl)-N-(2-pyridyl)amino]ethyl]-N-methylamino]ethyl]guanidine* (**26**). Yield: 50%, mp: >109 °C decomposed; ^1^H-NMR (CDCl_3_): δ ppm = 2.23 (s, 3H, N-CH_3_); 2.50 (m, 2H, CH_2_-S); 2.55 (m, 4H, 2(CH_2_-NCH_3_)); 3.15 (m, 2H, CH_2_-NH); 3.27 (m, 2H, CH_2_-NH); 3.58 (m, 4H, Thi-CH_2_-S, CH_2_-N); 4.72 (s, 2H, Ph-CH_2_-N); 6.46 (s, 1H, Thi5-H); 6.51–6.54 (m, 2H aromatic, Py5-H, Py3-H); 6.80 (br, 6H*, 2(NH), NC(NH_2_)_2_); 7.11 (t, *J*=9, 2H aromatic, 2-H); 7.22–7.24 (d, J=6, 2H aromatic, 3-H); 7.44 (m, 1H aromatic, Py4-H); 8.06 (d, 1H aromatic, Py6-H); MS: *m/z* (%) 584 (M^+^, 3), 121 (100); Anal. Calcd. for C_26_H_34_FN_11_S_2_・2C_4_H_6_O_6_・1.5H_2_O: C, 44.80; H, 5.42; N, 16.90. Found: C, 44.61; H, 4.91; N, 17.11.

*N-Cyano-N'-[2-[[(2-guanidino-4-thiazolyl)methyl]thio]ethyl]-N″-[3-[N-[2-[N-(4-methoxybenzyl)-N-(2-pyridyl)amino]ethyl]-N-methylamino]propyl]guanidine* (**27**). Yield: 45%, mp: 54–56 °C; ^1^H-NMR (CDCl_3_): δ ppm = 1.66 (m, 2H, CH_2_); 2.26 (s, 3H, N-CH_3_); 2.48 (m, 2H, CH_2_-S); 2.58 (t, *J* = 7, 2H, CH_2_-NCH_3_); 2.68 (t, *J* = 7, 2H, CH_2_-NCH_3_); 3.21 (m, 2H, CH_2_-NH); 3.29 (m, 2H, CH_2_-NH); 3.58 (s, 2H, Thi-CH_2_-S); 3.72 (t, *J* = 8, 2H, CH_2_-N); 3.76 (s, 3H, Ph-OCH_3_); 4.60 (s, 2H, Ph-CH_2_-N); 5.74 (br, 2H*, 2(NH)); 6.33 (S, 1H aromatic, Thi5-H); 6.43–6.44 (d, *J* = 8.5, 2H aromatic, Py5-H, Py3-H); 6.54 (br, 4H*, NC(NH_2_)_2_); 6.81–6.84 (m, 2H aromatic, 2-H); 7.10–7.13 (d, *J*=8,5, 2H aromatic, 3-H); 7.34–7.39 (m, 1H aromatic, Py4-H); 8.11–8.12 (d, J=3, 1H aromatic, Py6-H); MS: *m/z* (%) 624 (M^+^, 1), 121 (100); Anal. Calcd. for C_28_H_39_N_11_OS_2_・H_2_O: C, 53.60; H, 6.58; N, 24.50. Found: C, 53.81; H, 6.43; N, 24.41.

*N-Cyano-N'-[3-[N-[2-[N-(4-fluorobenzyl)-N-(2-pyridyl)amino]ethyl]-N-methylamino]propyl]-N″-[2-[[2-guanidino-4-thiazolyl)methyl]thio]ethyl]guanidine* (**28**). Yield: 35%, mp: oil; ^1^H-NMR (CDCl_3_): δ ppm = 1.67 (m, 2H ,CH_2_); 2.27 (s, 3H, N-CH_3_); 2.49 (t, 2H, CH_2_-S); 2.58 (t, 2H, CH_2_, NCH_3_); 2.73 (t, 2H, CH_2_-NCH_3_); 3.21 (m, 2H, CH_2_-NH); 3.32 (m, 2H, CH_2_-NH); 3.60 (s, 2H, Thi-CH_2_-S); 3.73 (m, 2H, CH_2_-N); 4.66 (s, 2H, Ph-CH_2_-N); 6.35 (s, 1H aromatic, Thi5-H); 6.41–6.43 (d, *J* = 9, 1H aromatic, Py5-H); 6.57 (t, *J* = 5, 1H aromatic, Py3-H); 6.80 (br, 6H*, 2(NH), NC(NH_2_)_2_); 6.99 (t, *J*=8.5, 2H aromatic, 2-H); 7.17 (m, 2H aromatic, 3H); 7.37–7.39 (m, 1H aromatic, Py4-H); 8.14 (d, 1H, Py6-H); MS: *m/z* (%) 598 (M^+^, 2), 121 (100); Anal. Calcd. for C_27_H_36_FN_11_S_2_: C, 53.80; H, 6.58; N, 24.50. Found: C, 53.81; H, 6.43; N, 24.40.

*N-Cyano-N'-[2-[[(2-guanidino-4-thiazolyl)methyl]thio]ethyl]-N″-[4-[N-[2-[N-(4-methoxybenzyl)-N-(2-pyridyl)amino]ethyl]-N-methylamino]butyl]guanidine* (**29**). Yield: 60%, mp: hygroscopic foam; ^1^H-NMR (CDCl_3_): δ ppm = 1.36–1.44 (m, 4H, 2(CH_2_)); (see **25**); MS: *m/z* (%) 624 (M^+^, 1), 121 (100); Anal. Calcd. for C_29_H_41_N_11_OS_2_・1.5H_2_O: C, 53.50; H, 6.81; N, 23.70. Found: C, 53.42; H, 6.51; N, 24.01.

*N-Cyano-N'-[4-[N-[2-[N-(4-fluorobenzyl)-N-(2-pyridyl)amino]ethyl]-N-methylamino]butyl]-N″-[2-[[2-guanidino-4-thiazolyl)methyl]thio]ethyl]guanidine* (**30**). Yield: 60%, mp: >137 °C decomposed; ^1^H-NMR (CDCl_3_): δ ppm = 1.49–1.58 (m, 2H, CH_2_); (see **28**); MS: *m/z* (%) 612 (M^+^, 2), 229 (100), 109 (38); Anal. Calcd. for C_28_H_38_FN_11_S_2_·1.5C_20_H_18_O_8_・2H_2_O: C, 56.80; H, 5.66; N, 12.60. Found: C, 56.40; H, 5.30; N, 12.51.

*N-Cyano-N'-[5-[N-[2-[N-(4-fluorobenzyl)-N-(2-pyridyl)amino]ethyl]-N-methylamino]pentyl]-N″-[2-[[2-guanidino-4-thiazolyl)methyl]thio]ethyl]guanidine* (**31**). Yield: 29%, mp: 103–105 °C; ^1^H-NMR (CDCl_3_): δ ppm = 1.23 (m, 2H, CH_2_); 1.35 (m, 4H, 2(CH_2_)); (see **26**); MS: *m/z* (%) 626 (M^+^, 3), 154 (100); Anal. Calcd. for C_29_H_40_FN_11_S_2_・3C_4_H_6_O_6_·0.5H_2_O: C, 45.40; H, 5.48; N, 14.20. Found: C, 45.12; H, 5.32; N, 14.19.

*N-Cyano-N'-[2-[[(2-guanidino-4-thiazolyl)methyl]thio]ethyl]-N″-[6-[N-[2-[N-(4-methoxybenzyl)-N-(2-pyridyl)amino]ethyl]-N-methylamino]hexyl]guanidine* (**32**). Yield: 29%, mp: >52 °C decomp.; ^1^H-NMR (CDCl_3_): δ ppm = 1.27 (m, 4H, 2(CH_2_)); 1.42 (m, 2H, CH_2_); (see **27**); MS: *m/z* (%) 652 (M^+^, 12), 121 (100); Anal. Calcd. for C_31_H_45_N_11_OS_2_・0.5H_2_O: C, 56.31; H, 7.01; N, 23.30. Found: C, 56.42; H, 7.17; N, 23.31.

*N-Cyano-N'-[6-[N-[2-[N-(4-fluorobenzyl)-N-(2-pyridyl)amino]ethyl]-N-methylamino]hexyl]-N″-[2-[[2-guanidino-4-thiazolyl)methyl]thio]ethyl]guanidine* (**33**). Yield: 61%, mp: 75–77 °C decomp.; ^1^H-NMR (CDCl_3_): δ ppm = 1.26 (m, 4H, 2(CH_2_)); 1.43 (m, 2H, CH_2_); (see **28**); MS: *m/z* (%) 639 (M^+^, 9), 229 (100), 109 (88); Anal. Calcd. for C_30_H_42_FN_11_S_2_・3C_4_H_6_O_6_·0.25H_2_O: C, 46.10; H, 5.57; N, 14.10. Found: C, 45.81; H, 5.62; N, 14.62.

*N-Cyano-N'-[7-[N-[2-[N-(4-fluorobenzyl)-N-(2-pyridyl)amino]ethyl]-N-methylamino]heptyl]-N″-[2-[[2-guanidino-4-thiazolyl)methyl]thio]ethyl]guanidine* (**34**). Yield: 24%, mp: 118–120 °C; ^1^H-NMR (CDCl_3_): δ ppm = 1.24 (m, 6H, 3(CH_2_)); 1.42 (m, 2H, CH_2_); (see **28**); MS: *m/z* (%) 654 (M^+^, 2), 91 (100); Anal. Calcd. for C_31_H_44_FN_11_S_2_·1.5C_20_H_18_O_8_・2H_2_O: C, 57.71; H, 5.96; N, 12.11. Found: C, 57.61; H, 5.51; N, 12.42.

*N-Cyano-N'-[3-[N-[2-[N-(4-methoxybenzyl)-N-(2-pyridyl)amino]ethyl]-N-methylamino]propyl]-N″-[2-[5-(dimethylaminomethyl)furfuryl]thio]-ethyl]guanidine* (**35**). Yield: 38%, mp: 74–76 °C; ^1^H-NMR (CDCl_3_): δ ppm = 1.68 (q, 2H, CH_2_); 2.23 (s, 6H, N(CH_3_)_2_); 2.28 (s, 3H, N-CH_3_); 2.49 (t, 2H, CH_2_-S); 2.56–2.65 (m, 4H, 2(CH_2_-NCH_3_)); 3.23–3.30 (m, 4H, 2(CH_2_-NH)); 3.40 (s, 2H, Fur-CH_2_-S); 3.67 (s, 2H, Fur-CH_2_-N); 3.75 (t, *J* = 8, 2H, CH_2_-N); 3.78 (s, 3H, Ph-OCH_3_); 4.63 (s, 2H, Ph-CH_2_-N); 6.11–6.12 (q, *J* = 4, 2H aromatic, Fur3-H, Fur4-H); 6.44–6.47 (d, *J* = 8.5, 1H aromatic, Py5-H); 6.55 (m, 1H aromatic, Py3-H); 6.55 (br, 1H*, NH); 6.83–6.85 (d, *J* = 8.5, 2H aromatic, 2-H); 6.83–6.85 (br, 1H*); 7.12–7.15 (d, *J* = 8.5, 2H aromatic, 3-H); 7.38 (m, 1H aromatic, Py4-H); 8.12–8.13 (d, *J* = 3, 1H aromatic, Py6-H); MS: *m/z* (%) 595 (M^+^, 4), 121 (100); Anal. Calcd. for C_31_H_44_N_8_O_2_S·3C_4_H_6_O_6_·0.5H_2_O: C, 49.10; H, 6.03; N, 10.71. Found: C, 48.71; H, 6.26; N, 11.22.

### 3.2. Pharmacology

#### 3.2.1. Histamine H_1_ Receptor Antagonist Activity on the Isolated Guinea Pig Ileum

H_1_ receptor antagonist activity was determined from isotonically recorded (load 10 mN) cumulative concentration-response curves as described using histamine dihydrochloride (0.01–3 µM) as the reference agonist [31]. Whole segments of proximal ileum were mounted in an organ bath containing 20 mL of Tyrode solution ([mM] NaCl 137, KCl 2.7, NaH_2_PO_4_ 4.2, NaHCO_3_ 11.9, CaCl_2_ 1.8, MgCl_2_ 1.0, D-glucose 5.6) containing 0.1 µM atropine, aerated with 95% O_2_/5% CO_2_, bath temperature 37 °C. The compounds were tested at different concentrations with at least four experiments for each concentration. Determination of H_1_ receptor antagonism at low concentrations (≤10^−8^ M) of the potential antagonists required a longer incubation period up to 20 min in order to achieve equilibrium states; at higher concentrations periods of 10–15 min were sufficient. p*K*_B_ values (mean of at least three independent experiments) were calculated from the expression p*K*_B_ = −log [antagonist] + log (concentration ratio–1) as the compounds produced a dose-dependent depression of the concentration-response curves [32].

#### 3.2.2. Histamine H_2_ Receptor Antagonist Activity on the Isolated Guinea Pig Right Atrium

The investigations for H_2_ receptor antagonism followed the procedure described by Black *et al.* [33] with minor modifications. In brief, male guinea pigs (350–400 g) were killed by a blow on the head and exsanguinated. Right atria were rapidly removed, attached to a tissue holder in an organ bath (32.5 °C) containing 20 mL of Krebs-Henseleit solution, containing [mM] NaCl 118, NaHCO_3_ 25, KCl 4.7, KH_2_PO_4_ 1.2, CaCl_2_ 2.5, MgSO_4_ 1.6, glucose 6.2, gassed with 95% O_2_/5% CO_2_, bath temperature 32.5 °C. The antagonistic potency was determined from isometrically recorded cumulative concentration-response curves using histamine dihydrochloride (0.1–10 µM) as the reference substance [34]. The incubation period was generally 30 min for the antagonists. For pharmacological screening, 2–5 experiments were carried out for each antagonist at one or two concentrations (Table 1) and p*K*_B_ values were calculated as above.

## 4. Conclusions

Starting from lead compound **1** according to the working hypothesis that the incorporation of a mepyramine-like structure linked via a six-membered-spacer with a cyanoguanidine instead of urea as a polar group and combined with a tiotidine-like partial structure, significantly improved H_1_R antagonist activity and restored pronounced H_2_R antagonism. However, the affinity at both receptors should be further increased with respect to the development of combined H_1_R/H_2_R antihistamine as potential pharmacotherapeutic agents. Generally, pharmacological hybrids harbour the potential of improving the pharmacokinetic properties compared to single drugs with different half lives. Further progress in structural optimization seems possible through selection of other H_1_R and H_2_R pharmacophoric moieties known to possess histamine receptor subtype affinity. Nevertheless, this has to be proven experimentally, because the mutual affinity-conferring or -enhancing contribution of the respective substructures cannot be predicted on the basis of the structure-activity relationships available so far. Different routes of administration are conceivable, for example, topical applications of combined H_1_R/H_2_R antagonists in dermatology or systemic administration for premedication in anaesthesia and surgery or for the prevention of hypersensitivity reactions to drug treatment, e.g., in cancer chemotherapy. This has to be taken into consideration with respect to optimization of the physicochemical properties.
